# Mechanical Forces Induce Changes in VEGF and VEGFR-1/sFlt-1 Expression in Human Chondrocytes

**DOI:** 10.3390/ijms150915456

**Published:** 2014-09-01

**Authors:** Rainer Beckmann, Astrid Houben, Mersedeh Tohidnezhad, Nisreen Kweider, Athanassios Fragoulis, Christoph J. Wruck, Lars O. Brandenburg, Benita Hermanns-Sachweh, Mary B. Goldring, Thomas Pufe, Holger Jahr

**Affiliations:** 1 Department of Anatomy and Cell Biology, Rheinisch–Westfälische Technische Hochschule (RWTH) Aachen University, 52074 Aachen, Germany; E-Mails: rbeckmann@ukaachen.de (R.B.); astrid.houben@ukmuenster.de (A.H.); mtohidnezhad@ukaachen.de (M.T.); nkweider@ukaachen.de (N.K.); afragoulis@ukaachen.de (A.F.); cwruck@ukaachen.de (C.J.W.); lbrandenburg@ukaachen.de (L.O.B.); tpufe@ukaachen.de (T.P.); 2 Institute of Experimental Musculoskeletal Medicine, University Muenster, 48149 Muenster, Germany; 3 Department of Orthopaedic Surgery, RWTH Aachen University, 52074 Aachen, Germany; 4 Department of Pathology, RWTH Aachen University, 52074 Aachen, Germany; E-Mail: bhermanns@ukaachen.de; 5 Research Division, Hospital for Special Surgery, Weill Cornell Medical College, New York, NY 10021, USA; E-Mail: goldringm@hss.edu

**Keywords:** VEGF-A, VEGFR-1/FLT-1, sVEGFR-1/FLT-1, cyclic stretch, strain, human chondrocyte, C-28/I2

## Abstract

Expression of the pro-angiogenic vascular endothelial growth factor (VEGF) stimulates angiogenesis and correlates with the progression of osteoarthritis. Mechanical joint loading seems to contribute to this cartilage pathology. Cyclic equibiaxial strains of 1% to 16% for 12 h, respectively, induced expression of VEGF in human chondrocytes dose- and frequency-dependently. Stretch-mediated VEGF induction was more prominent in the human chondrocyte cell line C-28/I2 than in primary articular chondrocytes. Twelve hours of 8% stretch induced VEGF expression to 175% of unstrained controls for at least 24 h post stretching, in promoter reporter and enzyme-linked immunosorbent assay (ELISA) studies. High affinity soluble VEGF-receptor, sVEGFR-1/sFlt-1 was less stretch-inducible than its ligand, VEGF-A, in these cells. ELISA assays demonstrated, for the first time, a stretch-mediated suppression of sVEGFR-1 secretion 24 h after stretching. Overall, strained chondrocytes activate their VEGF expression, but in contrast, strain appears to suppress the secretion of the major VEGF decoy receptor (sVEGFR-1/sFlt-1). The latter may deplete a biologically relevant feedback regulation to inhibit destructive angiogenesis in articular cartilage. Our data suggest that mechanical stretch can induce morphological changes in human chondrocytes *in vitro*. More importantly, it induces disturbed VEGF signaling, providing a molecular mechanism for a stress-induced increase in angiogenesis in cartilage pathologies.

## 1. Introduction

In joint cartilages, chondrocytes are constantly deformed as a result of loading due to normal daily activities. Guilak *et al.* estimated compression of chondrocytes resulting from physiological loading to be approximately 20% [1]. Normal physiological loading is generally regarded as a prerequisite for the maintenance of proper articular joint functioning, while injurious loading can lead to cartilage degeneration [2]. Other forms of mechanical stimulation like mechanical stretch also elicit a response in primary bovine chondrocytes [3,4]. In normal, healthy human chondrocytes, cyclic stretch has been reported to be anabolic [5], while others report differentiation stage-dependent detrimental effects in osteoarthritic cells [6]. Excessive mechanical stress causes deterioration of the cartilage metabolism through induction of catabolic factors, including matrix metalloproteinases (MMPs) [4,7,8].

However, mechanical loading is an important environmental factor that regulates articular cartilage homeostasis and influences the biosynthesis of matrix components *in vivo* [9,10]. Mechanical overload induces cartilage destruction and secondary osteoarthritis [11] as evident from acute traumatic injury, abnormal weight bearing (*i.e.*, obesity [12,13]) or altered joint geometries [14]. Being composed of a network of extracellular matrix components and scattered chondrocytes, healthy mature articular cartilage is essentially devoid of vasculature [15]. In osteoarthritis (OA), pro-angiogenic factors are produced by chondrocytes [16] and expression of vascular endothelial growth factor (VEGF) has been shown in the superficial zone of the tibial plateau in OA patients with degenerative changes, but not in healthy cartilage [17]. Pro-angiogenic stimuli alone might be insufficient to overcome the resistance of normal articular cartilage to neovascularization, which is at least partly due to its matrix composition [18]. Cartilage from patients with OA is also less able to remain avascular than healthy cartilage [19]. Abnormal mechanical stress might “awaken” adult chondrocytes to produce VEGF in order to increase their catabolic activity [7]. While VEGF plays an essential role in cartilage vascularization and endochondral bone development [20], Wong *et al.* [21] also showed that VEGF is significantly up-regulated by cyclic tension and hydrostatic pressure in chondrocytes. Not surprisingly, recent investigations also revealed higher expression levels of VEGF and its receptors in diseased cartilage, such as in OA and rheumatoid arthritis (RA) [16,22,23,24]. Pufe *et al.* [25] further showed that VEGFA significantly increased matrix metalloproteinase (MMP) levels in cultured immortalized human chondrocytic C-28/I2 cells. Nevertheless, the precise mechanism by which VEGF might be involved in the pathogenesis of OA is not clearly understood.

Being composed of a network of extracellular matrix components and scattered chondrocytes, healthy mature articular cartilage is essentially devoid of vasculature [15]. The mechanisms by which articular cartilage might be maintained as avascular have not been fully clarified. Chondrocyte hypertrophy is one of the key physiological processes involved in the longitudinal growth of long bones, but also in the development of OA [17,26]. Hypertrophy is accompanied by an up-regulation of collagen X, MMPs, and VEGF [26].

The VEGF family comprises at least seven members [27] of which VEGF-A, or simply VEGF [28], is the founding member encoded by the *VEGF* gene and thought to be of singular importance [29]. Hypoxia facilitates the binding of hypoxia-inducible factor 1 (HIF-1) to the hypoxia responsive element (HRE) in the 5' promoter region of the *VEGF* gene to induce its expression [30]. While VEGF binds to all VEGF receptors, its affinity to VEGFR-1 (or fms-like tyrosine kinase-1, Flt-1) is 10-fold higher than to VEGFR-2 (or kinase domain region (KDR)/fetal liver kinase-1, Flk-1) [31,32]. Therefore, VEGFR-1 is usually considered to act as a sink for VEGF isoforms [33,34]. Alternative splicing of VEGFR-1 also generates a soluble form, sVEGFR-1 (synonym: sFlt-1), which acts as an extracellularly circulating decoy receptor to negatively regulate VEGF activity [34]. Although expression of VEGF is pro-angiogenic and a potential challenge for physiologically avascular tissues, little is known about its induction or about the regulation of its receptors, like sVEGFR-1.

With the present study, we aimed to investigate the intersection between VEGF signaling pathways and mechanosensation in chondrocytes. Specifically, we wondered if VEGF, its high affinity receptor VEGFR-1 and its endogenous inhibitor sVEGFR-1 are differentially regulated by different magnitudes of stretch.

## 2. Results and Discussion

### 2.1. Results

First, we subjected C-28/I2 cells and primary chondrocytes to a 12 h cyclic stretching regime using cyclic square waveforms. We correlated the amount of relative stretching of the BioFlex^®^ silicone bottom membrane and its frequency to VEGF expression in these cells: a range of 1% to 16% of stretch at 0.5 or 1 Hz, respectively, was evaluated and normalized to the non-stretched controls.

**Figure 1:**
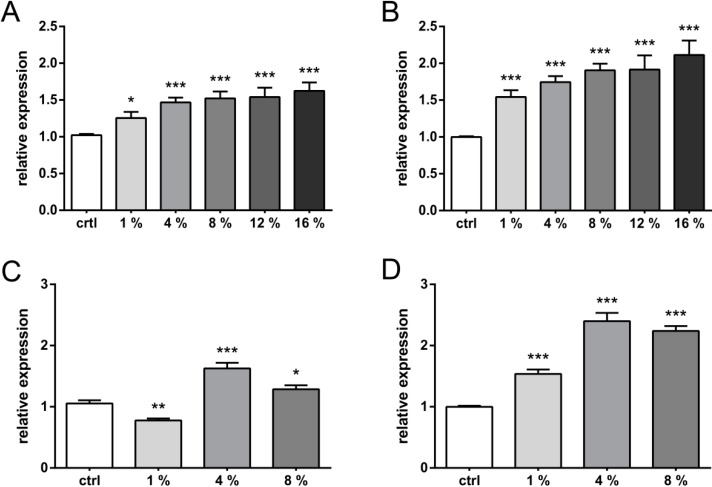
Strain-dependent vascular endothelial growth factor (VEGF) expression in C-28/I2 cells. Chondrocytes were strained in a FX-4000T system for 12 h. Using square waveforms and 0.5 Hz at 0%, 1%, 4%, 8%, 12% and 16% of strain (**A** and **B**, respectively), VEGF expression was quantified immediately (*t*_0_) (**A** and **C**, respectively) or 24 h post stretching (**B** and **D**, respectively) using a VEGFA-specific enzyme-linked immunosorbent assay (ELISA). The experiment was repeated at 1 Hz using only 0%, 1%, 4%, and 8% of strain (**C** and **D**, respectively). All values are normalized to the unstrained, parallel controls (set to 1). *, **, and *** indicate significance levels of *p* ≤ 0.05, *p* ≤ 0.01, and *p* ≤ 0.001 respectively, *n* = 5.

Using square waveforms, low frequency (0.5 Hz) stimulation strain dose-dependently induced VEGF secretion to 160% in C-28/I2 cells (Figure 1A) immediately after the stretching regime at maximal elongation (*i.e.*, 16%). Under these conditions, modest stretching of only 1% already induced VEGF secretion by 25% and 4% stretch by 50%, respectively.

Stretch-induced VEGF secretion continued in resting cells: 24 h after the cyclic stretching, strain-dependent VEGF secretion reached 220% (*i.e.*, 206 pg/mL) of the control level at 16% elongation and increased to more than 150% in the 1% stretch condition (Figure 1B). Doubling the stretch frequency to 1 Hz initially suppressed VEGF secretion at very low elongation rates (*i.e.*, 1% stretch), while 4% of stretch resulted in a similar, about 50%, induction of VEGF secretion as compared to 0.5 Hz. Surprisingly, while 0.5 Hz showed an almost linear dose-response between strain levels and VEGF secretion, VEGF secretion at 1Hz dropped already at 8% of stretch immediately after stretching (Figure 1C). Low end stretch of 1% did also not further induce VEGF secretion upon non-stretched incubation under these conditions. In contrast, VEGF secretion reached 240% of the control level upon 24 h of non-stretched incubation at only 4% stretch, indicating a continuous secretion of this pro-angiogenic cytokine beyond the point of direct straining (Figure 1C *vs.* 1D). This is also 29% more than at the highest stretch level of 16% at 0.5 Hz and about 65% more than at the 4% stretch at 0.5 Hz. Doubling the stretch level to 8% did not further increase the VEGF concentration in the culture medium. Rather, a relative drop of about 25% was observed, roughly mirroring the VEGF secretion pattern directly after stretching. At 1 Hz, VEGF secretion was maximal at 4% of stretch independent of the timing.

**Figure 2:**
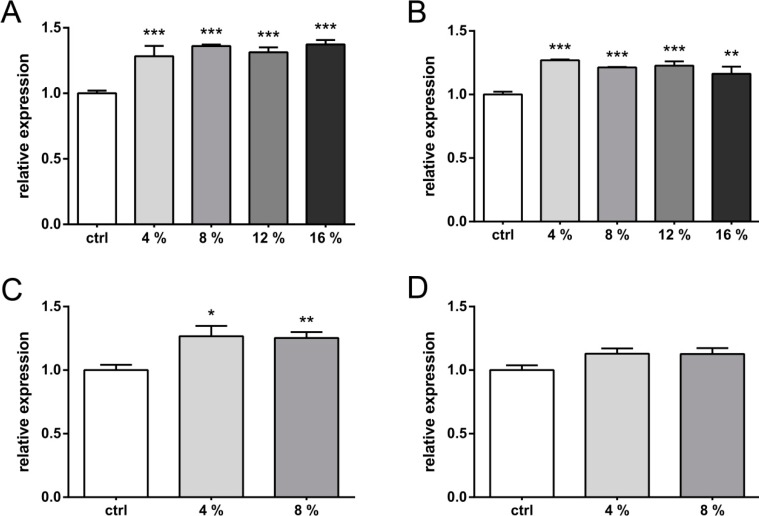
Strain-dependent VEGF expression in primary chondrocytes. Cells were strained for 12 h with cyclic waveforms and a variety of relative strains. VEGF expression was analyzed directly after straining (*t*_0_) (**A** and **C**, respectively) or 24 h post stretching (**B** and **D**, respectively). The figures show data obtained with square waveforms at 0.5 Hz (**A**,**B**) and 1.0 Hz (**C**,**D**). Relative percentages of stretching are indicated (*i.e.*, 4%, 8%, 12% or 16%, respectively). Unstrained controls (ctrl) were used to normalize data (set to 1). *, **, and *** indicate significance levels of *p* ≤ 0.05, *p* ≤ 0.01, and *p* ≤ 0.001 respectively, *n* = 3.

In primary articular chondrocytes (Figure 2), the strain-dose response with square waveforms at 0.5 Hz was less linear than in C-28/I2 cells. While the VEGF secretion ascended in C-28/I2 with increasing stretch levels between 1% and 16%, in primary chondrocytes a plateau was reached at 4% of stretch immediately after stretching (Figure 2A). Between 4% and 16% of stretch, VEGF secretion appeared relatively independent of the applied strain level also upon 24 h of non-stretched incubation (Figure 2B). VEGF secretion in primary cells at 4% reached 128% and was thus virtually identical to the level in C-28/I2, immediately after straining (Figure 2A *vs.* 1A). Increasing the frequency to 1 Hz did not enhance VEGF secretion in primary chondrocytes (Figure 2A *vs.* 2C). No significant elevation of VEGF levels was found after 24 h of non-stretched incubation (Figure 2D).

**Figure 3:**
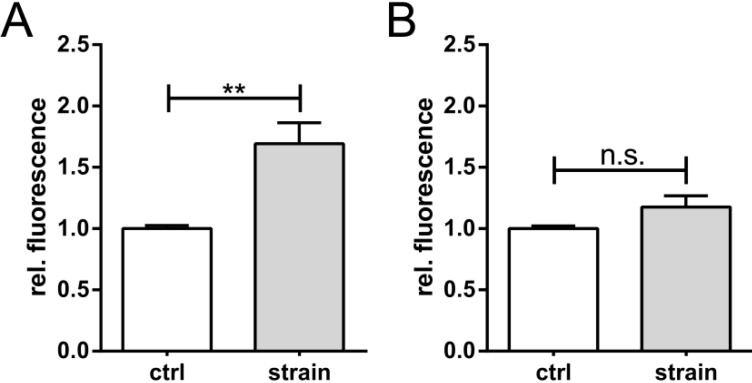
Stretch-mediated activation of VEGF promoter activity. Dual luciferase reporter assays were used to screen VEGF promoter activation upon stretching. C-28/I2 chondrocytes were transfected with a VEGF-promoter specific reporter construct (pVEGF-KpnI), expressing inducible Firefly luciferase, and a constitutive active *Renilla* luciferase for normalization purposes. Cells were stretched (8%, 0.5 Hz, 12 h square waveforms (**A**) and the ratio of Firefly-to-*Renilla* signals is shown. The unstrained control (ctrl) is set to 100% (*i.e.*, 1); HRE activity (**B**) was measured using a Hypoxia Responsive Element (HRE) reporter construct (pGL3-HRE). Unstrained controls (ctrl), stretched cells (strain). ** indicates *p* ≤ 0.01, n.s. = not significant; *n* = 5.

Next, we used dual-luciferase-assays to verify stretch-induced VEGF biosynthesis; stretching human chondrocytes induced the VEGF promoter activity in these cells to 175% of the control level (Figure 3A). In comparison, upon 8% stretching at 0.5 Hz, VEGF secretion increased to 150% (Figure 1A).

Binding of HIF-1α to the HRE in the 5' promoter region of the *Vegf* gene can induce its expression [30]. For that reason, we used a pGL3: HRE-luc vector as a negative control. Under normoxic conditions, when HIF-1α is unstable, stretch alone is not able to activate transcription of this reporter (Figure 1B). Our data confirm a stretch-specific, HIF-1α independent, induction of *Vegf* transcription in human chondrocytes.

**Figure 4:**
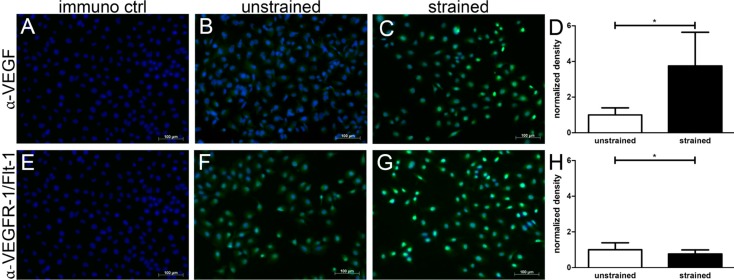
Expression of VEGF and VEGFR-1 in human chondrocytes.Representative immunofluorescent staining of VEGF and VEGFR-1 in C-28/I2 chondrocytes. Cells were strained for 12 h with cyclic square waveforms at 0.5 Hz and 8% relative stretching. Green Alexa Fluor^®^ 488-specific signals for VEGF (**top row**) and VEGFR-1 (**bottom row**) are shown, while nuclei appear in blue (bisbenzimid). Staining control without primary antibody (immuno ctrl: **A**,**E**); unstrained cells (**B**,**F**), strained (*i.e.*, stretched) cells (**C**,**G**). Scale bars represent 100μm. Signal intensity of the anti VEGF staining (**D**) and VEGFR-1/Flt-1 (**H**) was quantified by ImageJ. Signal intensity of unstrained cells was set to 1. * indicates *p* ≤ 0.05, *n* = 3.

Finally, we confirmed the induction of VEGF expression on cellular level in human C-28/I2 chondrocytes. Upon 12 h of 8% cyclic square waveforms stretching at 0.5 Hz, increased green fluorescent signals indicated the induction of VEGF biosynthesis (Figure 4C *vs.* 4B). Non-strained control cells revealed only very faint VEGF signals, indicating a very modest basal expression level (Figure 4B). Controls lacking the primary antibody were only positive for nuclear counterstaining (blue, Figure 4A). VEGF has a predominant high affinity receptor: VEGFR-1. Therefore, we next looked into the strain-dependency of its expression, too. In contrast to its prime ligand, VEGFR-1-specific immunosignals were already quite prominent in non-stretched cells (Figure 4E) and appeared to be cytoplasmic as well as nuclear. Surprisingly, 8% of stretching hardly decreased VEGFR-1-specific staining intensity in these cells (Figure 4F).

**Figure 5:**
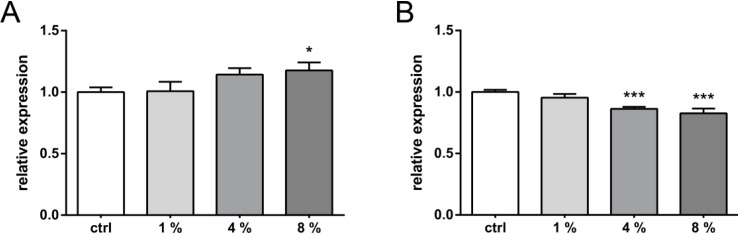
Stretch dependency of sVEGFR-1 expression in C-28/I2. Cells were stretched for 12 h with cyclic waveforms and a variety of relative strains. Expression of soluble decoy VEGF receptor: sVEGFR-1 was analyzed directly after straining (*t*_0_) (**A**) or 24 h post straining (**B**), using cyclic square waveform at 0.5 Hz. Relative percentages of stretching are indicated (*i.e.*, 1%, 4% or 8%, respectively). Unstrained controls (ctrl) were used to normalize data (set to 1). * and *** indicate significance levels of *p* ≤ 0.05 and *p* ≤ 0.001 respectively, *n* = 3.

VEGFR-1 is considered to be a sink for VEGF isoforms and alternative splicing can give rise to its soluble form, sVEGFR-1. We therefore postulated that the latter may act as a potential extracellular decoy receptor to inhibit VEGF-induced angiogenesis in cartilage, and aimed at quantifying the amount of strain-induced sVEGFR-1 secretion. Using square cyclic waveforms at 0.5 Hz, sVEGFR-1 secretion increased only about 20% immediately after 8% of stretching. Neither 1% nor 4% stress significantly increased its secretion level (Figure 5A). Of note, after 24 h, the trend in strain-induced sVEGFR-1 secretion reversed and revealed suppression for ≥4% of initial stretching (Figure 5B).

**Figure 6:**
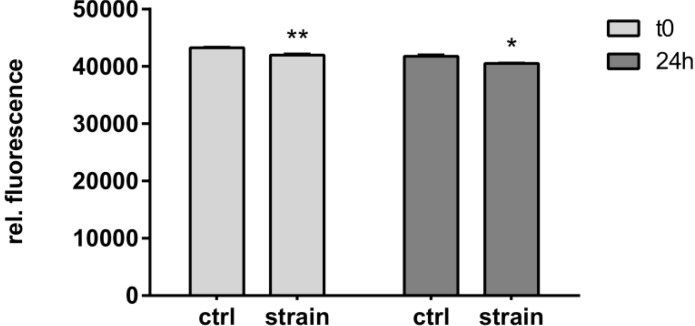
Effect of strain on cell viability. C-28/I2 chondrocytes were stretched (8%) for 12 h with cyclic square waveforms at 1 Hz. Cell viability was assessed using CellTiter Blue^®^ directly after straining (*t*_0_) or 24 h later, and is expressed as relative fluorescent signal (560 nm/590 nm ratio). Unstrained controls (ctrl), stretched cells (strain). * and ** indicate significance levels of *p* ≤ 0.05 and *p* ≤ 0.01, respectively, *n* = 3.

To exclude strain-induced changes in C-28/I2 viability, we determined the metabolic activity of cells stretched with 8% at 1Hz, which resulted in a reduced VEGF expression as compared to 4% stretch (Figure 1C,D). Using cyclic square waveforms only marginally, but significantly, reduced metabolic activity in C-28/I2 cells (Figure 6). The same trend was seen immediately after stretching as well as 24 h later.

**Figure 7:**
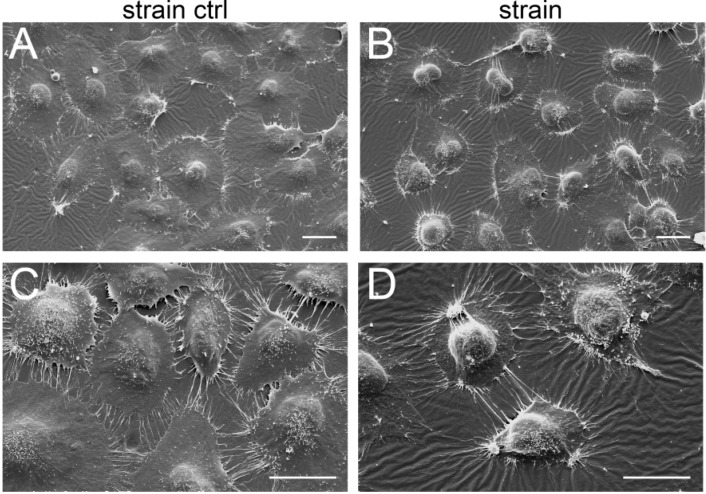
Morphological changes in stretched chondrocytes. C-28/I2 cells were stretched (8%) for 12 h using cyclic square waveforms at 0.5 Hz and immediately fixed on the silicone membrane. Shown are representative scanning electron micrographs of unstrained cells (strain ctrl: **A**,**C**) and stretched cells (strain: **B**,**D**). Scale bars represent 25 μm. *n* = 3.

Using scanning electron microscopy (SEM), we confirmed the typical polygonal morphology of C-28/I2 cells. Strained cells (Figure 7B,D) showed a more prominently pronounced nucleus as compared to non-stretched cells (Figure 7A,C). In addition, they revealed a higher averaged nucleus/cytoplasm ratio as the cytoplasm of strained cells appeared more condensed and cells revealed characteristic knot-like condensations at the cellular periphery (Figure 7D). The latter could be caused by adhesive structures. Furthermore, the cellular protrusions (*i.e.*, filopodia) appear more directed, possibly aligned in the direction of the strain. Stretched cells also reveal characteristic knot-like condensations at the cellular periphery (Figure 7D).

### 2.2. Discussion

Mechanical forces are believed to substantially contribute to the onset and progression of osteoarthritis [11,35,36], affecting about 2% of the population [37]. Expression of pro-angiogenic vascular endothelial growth factor (VEGF) stimulates angiogenesis and also correlates with OA [38]. This suggests an active role of this cytokine in this pathogenesis and has been confirmed in a murine model [37]. In the present study, we showed that cyclic stretch dose- and frequency-dependently induced VEGF expression in primary human articular chondrocytes as well as in a chondrocytic cell line. We did not investigate mRNA expression of hypertrophic markers, but rather focused on the expression of VEGF signaling molecules at the protein level.

A 7% cyclic tensile strain of 0.5 Hz for 24 h did affect MMP-13 expression in rat chondrocytes [39], which is in line with the observed VEGF induction in our experiments, as MMP-13 and VEGF are both reported to be Runx-2 dependent [40]. Stretching chondrocytes at 0.5 Hz showed an almost linear dose-response between strain levels and VEGF secretion. Intriguingly, at 1 Hz, 4% of stretching induced the largest VEGF secretion (Figure 1C,D). Alternative splicing of the primary VEGF pre-mRNA transcript produces at least five different isoforms of which VEGF-121 is totally released into the supernatant, whereas 70% of VEGF 165 may remain trapped into the extra-cellular matrix (ECM) [41]. To our knowledge, only two other studies have investigated the effect of strain frequency on skeletal cells before: one study by our group [42] reported frequency-dependent VEGF-121 and -165 syntheses in tendon fibroblasts at 1 Hz, but not at 0.5 Hz. The other study [43] used ROS17/2.8 rat osteosarcoma cells. Using our type of straining device and an ELISA assays from the same company, Faure *et al.* further found that low frequencies (≤0.25 Hz) increased VEGF secretion into the supernatant (1.5- and 1.2-fold, respectively), while higher frequencies (≥2.5 Hz) had no effect or decreased VEGF release, which is thus negatively correlated with the stretching frequency. The authors hypothesized that relaxed and pulse mode-driven alternative start codon usage shifts VEGF isoform expression in a stress-fiber dependent manner. Intriguingly, both VEGF and mechanical stimuli regulate stress fiber formation in skeletal cells [44]. As cytoskeletal integrity is a prerequisite to mechanosensitivity [45], we speculate that not only frequency, but also the magnitude of stretching can influence VEGF release in a similar way.

Our stretch-mediated VEGF release is rather moderate. Others also reported plenty of evidence across cell types suggesting that even moderate changes in VEGF levels can be biologically meaningful with respect to stimulating angiogenesis [43,46,47], and that 10% cyclic stretch at 1 Hz can increase sprouting angiogenesis [48]. Interestingly, cyclic uniaxial stretch apparently is a pro-angiogenic stimulant, like VEGF itself, and a synergistic effect between stretching and VEGF was found for especially the low, not high, cytokine concentrations. A not even 2-fold increase in VEGF secretion (173.4 *vs.* 275.73 pg/mL) by keratinocytes was found to stimulate local blood vessel formation [49]. Stretching elicited a similar fold-change of VEGF secretion, with concentrations around 200 pg/mL, by chondrocytes. We therefore believe this may be sufficient to stimulate angiogenesis *in vivo*, too.

In the present study we showed that different types of chondrocytes release VEGF upon mechanical stimulation. *In vivo* mechanical information is delivered to chondrocytes through various types of stimuli (*i.e.*, compression, tension, and fluid-flow or piezoelectric currents) all characterized by various amplitudes and frequencies. One limitation of our study is that we cannot easily translate our results to forces *in situ*. We found that relatively low strains are sufficient to induce VEGF in chondrocytes in monolayer culture, while others have described detrimental effects not below 16% of stretch at 0.5 Hz in other cells [50].

VEGF expression is known to be up-regulated through activation of ERK signaling, which is inducible by mechanical stretch [51]. Within the VEGF 5'-flanking region, negative and positive stretch-response elements have been identified [52]. Analysis of the human VEGF gene promoter sequence also revealed several consensus transcriptional response elements like AP-1, AP-2 and GATA-6, which are known molecular targets of MAPK signaling [53]. Of note, 5% of cyclic strain at 1 Hz for 24 h induced ERK and activated a mechano-sensitive HIF-1α element in the VEGF promoter inducing VEGF and, subsequently, MMPs [54]. The latter may be a sign of “injurious” strain levels. In closely related skeletal cells, HIF1α *trans*-activates *Vegf* and mechanical strain also was shown earlier to induce both Hif1α and *Vegf* expression in related cell types [43]. Even normoxic myocardial cells under mechanical stress induced HIF1α, and subsequently VEGF [55]. Surprisingly, we could not demonstrate HRE activation by stretch, which makes HIF1α involvement in our experiments unlikely.

That 7.5% of stretch at 1 Hz in a FlexerCell system induced MMP13 expression in articular chondrocytes [56] is supportive of our notion that a hypertrophic pathway (*i.e.*, collagen X expression) may be activated by cyclic tension in our chondrocytes [57]. As key factors of mechanotransduction, NO donors may also contribute to the VEGF induction [58]. Likewise, VEGF receptor signaling also involves calcium release and MAPK activation [59], both of which are known to be regulated by stretch [60,61,62]. It would be interesting to study their contribution to VEGF signaling in future studies.

The strain-mediated VEGF release of primary chondrocytes and immortalized cells slightly differed; C-28/I2 cells are immortalized using SV40 large T antigen, which binds cellular proteins such as Rb and p53. Different p53 levels in primary chondrocytes and C-28/I2 cells may thus contribute to the differences in stretch responsiveness on short and long-term effects on VEGF [63]. The VEGF regulation by the p53 family members is complex and involves several transcription factors able to induce or repress VEGF in a cell context-dependent manner [63]. From a biological point of view, it does not make sense that a physiological process like daily joint loading induces pro-angiogenic factors like VEGF in a naturally avascular tissue, unless there is a feedback system to secondarily inhibit VEGF’s unwanted action. The high-affinity receptor sVEGFR-1 may inhibit unwanted angiogenesis in articular cartilage through abolishing stretch-induced VEGF action; sVEGFR-1 is significantly induced immediately after stretching, but suppressed 24 h after stretching when VEGF is still maximally induced. Our data thus suggest that stretch may contribute to triggering pathological angiogenesis in cartilage and prompt caution with respect to unphysiological joint loading. The negative regulation of VEGF signaling is exerted, at least in part, by the alternatively spliced soluble VEGFR1 variant and may be established to prevent VEGF from binding to VEGFR-1 or VEGFR2 [59]. Another interesting but yet unclear feature of VEGFR1 is that its different ligands (e.g., VEGFA, VEGFB) transduce distinct biological responses [59] and VEGFR1 might positively modulate VEGFR2 outputs. Whether these apparently opposing effects of VEGFR1 on VEGFR2 activity are strain-dependent and involved in chondrocyte differentiation at all also remains to be investigated.

Another limitation of our study is that we stretched monolayer cells, whereas the 3D *in situ* loading mainly involves compression that leads to nuclear deformation of a potentially different order of magnitude [64]. Despite our consistent responses, *in vitro* systems never accurately represent the *in vivo* situation. This limits the interpretation of our results towards the *in vivo* situation. An emerging pattern in many biological responses to VEGF is the contribution by more than one type of VEGF receptor, which shows the crucial role of balanced signaling.

Unstrained cells showed a typical polygonal morphology, while strained cells showed a more prominently pronounced, very round nucleus (Figure 7). In addition, cellular filopodia appeared to be more pronounced in strained cells. Using uniaxial cyclic tensile strain, Greiner *et al.* [65] showed that initially cell protrusions are uniformly formed around cells. Upon *de novo* syntheses of ventral actin stress fibers, these protrusions line up perpendicular to the direction of the strain using similar tension system and 2%–8% strain [66]. We applied biaxial strain, which may explain the radial orientation of the filopodia. To our knowledge, this is the first report of scanning electron micrographs of strained chondrocytes. *In vitro* applied strains caused only marginally decreased mitochondrial metabolic activity in C-28I2 cells, while cell death in articular chondrocytes might already be in process when VEGF is re-upregulated by stress triggers in OA cartilage *in vivo* [67].

## 3. Experimental Section

### 3.1. Cell Culture

Human chondrocytes used in this study comprise the C-28/I2 cell line [68], a well-established model that has been shown to phenotypically resemble articular chondrocytes [69], and primary human articular chondrocytes. For the latter, after local ethical approval and informed consent, cartilage was obtained from the femoral head region of a young, non-arthritic patient undergoing reconstruction surgery. Briefly, full thickness cartilage was harvested, chopped into pieces, washed and subsequently digested overnight in Ham’s F-12 medium as reported earlier [6]. The following day, harvested cell were cultured in DMEM/F-12 with 10% of heat-inactivated FCS and 1% Penicillin–Streptomycin–Amphotericin B. For all media and culture additives were purchased from Sigma–Aldrich (Steinheim, Germany). Experiments were performed at least in independent triplicates with, at least, technical duplicates. Sample sizes (*n*) are indicated per experiment in the legend.

### 3.2. Mechanical Stimulation

Upon seeding of 350,000 C-28/I2 cells and 300,000 primary chondrocytes on collagen type I (COL1) coated BioFlex^®^ six-well plates (FlexCell^®^ Int. Corp., Hillsborough, FL, USA), the mechanical stimulation of the chondrocytes was essentially performed as reported earlier by us [6], but with the following modifications: cells were statically pre-cultured in the BioFlex^®^ plates overnight (20–24 h) in 2.5 mL culture medium in a FlexCell Strain Unit FX-4000 to facilitate adherence to the flexible membrane. Cells were stretched in an incubator (37 °C, 5% CO_2_) as previously described [61] and the medium was exchanged immediately prior to stretching.

Briefly, a vacuum created under the six-well plates pulls the flexible-bottom membrane over a loading post, resulting in homogenous biaxial strain of up to 30% of substrate (*i.e.*, membrane) elongation. The used Flexercell^®^ Tension Plus™ unit FX-4000T setup has a minimum strain resolution capability of 0.7% to 20% elongation and delivers frequencies between 0.1 and 5 Hz with single cycle strain rates as high as 6.9 s^−1^ with infinitely small creep strains. The system delivers multi-cyclic strain rates (0.2–10 s^−1^) using square cyclic waveform of which we used cyclic stretch at a frequency of 0.5 and 1 Hz, respectively, for 12 h, and stretching between 1% and 16%, respectively. Wells with FlexStop™ posts to prevent pressure-induced stretching served as internal controls [6].

### 3.3. Biochemical Analyses

Two micro liters of culture medium were collected either immediately after the stretching regime (*t*_0_) or 24 h later. Samples were centrifuged (20,000 rcf, 5 min, 4 °C) and cell-free supernatants stored at −70 °C until further use.

Commercially available enzyme-linked immunosorbent assay (ELISA) kits were used to quantify the amount of hVEGF and hVEGFR-1 (synonym: Flt-1) (both from R&D Systems, Minneapolis, MN, USA) according to the manufacturer’s instructions. Briefly, medium samples and standards were incubated overnight at 4 °C in the pre-coated wells. Washing steps, incubation of target-specific, biotin-coupled secondary antibody, and detection antibody (horseradish peroxidase conjugated) was according to supplier’s guidelines. Enzymatic substrate (3,3',5,5'-tetramethylbenzidine) oxidation was quantified using a Infinite^®^ PRO reader (Tecan Germany GmbH, Crailsheim, Germnay) at 450 nm.

### 3.4. Cell Viability Assay

Cell viability assays were performed by using the CellTiter-Blue^®^ assay (Promega, Madison, WI, USA) according to the manufacturer’s description. The assay relies on the conversion of resazurin into the highly fluorescent resorufin by metabolically active cells. Briefly, upon applying cyclic 1/2 shine and square waveforms at 1 Hz and 8% stretching for 12 h, cells were washed once with 2 mL of 1× PBS and then incubated in culture medium for 24 h (controls), while the other cells were incubated with 0.2 mL of CellTiter-Blue^®^ reagent (1:5). After 60, 90 and 120 min, 0.1 mL of the reagent were transferred into 96-well plates for optical density measurements at 570 nm (reference: 590 nm) in the Infinite^®^ PRO reader against medium blanks.

### 3.5. Transient Transfections and Dual Luciferase Assays

Bacterial transformations and reporter plasmid DNA purifications were performed as described by Fragoulis *et al*. [70]. The 2.65 kb fragment of the human VEGF-A promoter region from plasmid pVEGF-*Kpn*I (ATCC^®^, American Type Culture Collection, Manassas, VA, USA) or the 25 bp human hypoxia-responsive element (HRE) was cloned into the *Mlu*I site of the mcs pGL3-basic luciferase reporter vector (Promega, Madison, WI, USA). Both promoter reporter plasmids were then transiently co-transfected with the constitutively active reporter *Renilla* luciferase vector phRL-TK (Promega, Madison, WI, USA) into chondrocytes. After overnight incubation, reporter cells were used in mechanical stimulation experiments on BioFlex^®^ plates (see above).

Quantification of stretch-induced promoter activation by dual luciferase assays was performed exactly as reported earlier by Fragoulis *et al.* [70].

### 3.6. Immunofluoresence

To visualize changes in VEGF and VEGFR-1 expression on cellular level, C-28/I2 chondrocytes were fixed for 30 min in a neutral 4% *v*/*v* formalin solution (Sigma–Aldrich) after a 12 h lasting 8% cyclic square waveform stretch regime at 0.5 Hz. Cells on pieces of dissect BioFlex^®^ silicone membranes were then permeabilized in 0.1% of Triton X-100 (Sigma–Aldrich) for 10 min and washed 3 times for 5 min in Tris-Buffer (25 mM, pH 7.4) essentially as described earlier [71]. Blocking occurred in 1.5% BSA in Tris-Buffer (10 min) prior to incubation with polyclonal anti-VEGF (Santa Cruz, sc-507; 1:50) and anti-VEGFR-1 (Santa Cruz, sc-316; 1:30) antibodies overnight at 4 °C. After washing, antigens were visualized using secondary Alexa-Fluor^®^ 488 labeled antibodies (Invitrogen, Karlsruhe, Germany; 1:250) and analyzed imbedded in Shandon Immu-Mount™ (Thermo Scientific, Pittsburgh, PA, USA). Nuclear counterstaining was performed with Hoechst 33258 (Molecular Probes^®^, Invitrogen, Karlsruhe, Germany; bisBenzimide pentahydrate, 1:200) in 1× neutral PBS. Pictures were made using AxioVision Release 4.8.2 (Carl Zeiss MicroImaging GmbH, Jena, Germany). Normalized fluorescent signal densities were measured as reported earlier [72] and according to [73]. Quantification was performed using ImageJ freeware [74].

### 3.7. Scanning Electron Microscopy (SEM)

Morphological changes in chondrocytes subjected to mechanical stimulation were assessed using SEM. Following a 12 h lasting 8% stretch regime at 1 Hz, C-28/I2 cells were immediately fixed in 3% *v*/*v* EM-grade glutaraldehyde (Sigma–Aldrich, G5882) in a 0.1 M Sörensen phosphate buffer (pH 7.4; 13 mM NaH_2_PO_4_ × H_2_O; 87 mM Na_2_HPO_4_ × 2H_2_O; all Sigma–Aldrich). Cells were cut out with their BioFlex^®^ silicone membrane and, upon routine dehydration in ethanol, critical point dried in liquid CO_2_ in a CPD 010 critical point dryer (BAL-TEC AG, Balzers, FL, USA). The samples were coated with 30 nm of gold in a SCD 500 sputter-coater (Leica Microsystems, Wetzlar, Deutschland) and analysed in a FEI/Philips XL 30 FEG ESEM (FEI, Frankfurt, Germany) in a high vacuum environment.

### 3.8. Statistics

Data are presented using GraphPad Prism 5 (La Jolla, CA, USA), showing the averages with standard deviation. Significance was tested using parametric, two-sided *t*-test using the average and significance was *p* ≤ 0.05.

## 4. Conclusions

Probably the most intriguing aspect of our study is that the high affinity receptor VEGFR-1 (Ftl-1) was less stretch-inducible than its ligand, VEGF-A. In addition, the secretion of the major VEGF decoy receptor, sVEGFR-1 (sFlt-1), appeared to be strain suppressible.

In recent years there has been an impressive development of clinical therapies aiming to enhance or suppress VEGF/VEGFR function. That even relatively short-term suppression of VEGFR function might lead to suppression of blood vessel formation is now obvious from studies in mouse models [60] and has significant potential as a means to fine-tune VEGF-based anti-OA therapies. Strain-induced sVEGFR-1 suppression in the long-term may therefore be detrimental to hyaline cartilage and suggests that even 4% relative elongation may be supra-physiological for chondrocytes.
